# The role and mechanisms of bone microenvironment regulators in osteoporosis: novel intervention strategies for addressing the challenges of aging

**DOI:** 10.3389/fendo.2026.1783422

**Published:** 2026-03-03

**Authors:** Hongyuan Yao, Yutao Cui, Peng Li, Shouye Sun, Chuangang Peng, Dankai Wu

**Affiliations:** 1 Traumatic Orthopedics, The Second Hospital of Jilin University, Changchun, China; 2 Department of Orthopedics, The First Affiliated Hospital of University of Science and Technology of China, Hefei, China

**Keywords:** aging, bone microenvironment, bone remodeling, osteoporosis, regulatory factors

## Abstract

Osteoporosis is an increasingly important global health concern, particularly in aging populations, with prevalence rising markedly after the age of 60. Age-related alterations in the bone microenvironment play a pivotal role in disrupting skeletal homeostasis. Regulators of the bone microenvironment contribute centrally to osteoporosis pathogenesis by modulating bone remodeling through multiple, intersecting mechanisms. Accumulating evidence indicates that aging is accompanied by reduced levels of protective factors, such as osteoprotegerin and bone morphogenetic proteins (BMPs), alongside increases in pro-resorptive mediators, including receptor activator of nuclear factor-κB ligand (RANKL), interleukin-6 (IL-6), and tumor necrosis factor-α (TNF-α). This shift favors osteoclastogenesis and impairs osteoblast function, ultimately accelerating bone loss and increasing the risk of fragility fractures and disability. In this review, we synthesize current evidence on bone microenvironment regulatory factors in osteoporosis, with emphasis on their roles in bone remodeling and downstream cellular signaling pathways. We further discuss emerging intervention strategies that target these regulators to preserve or restore bone health in older adults. By clarifying age-associated microenvironmental changes and the interactions among key regulatory factors, this review aims to identify promising therapeutic targets and provide a conceptual framework to support osteoporosis prevention and treatment in the context of global population aging.

## 1 Introduction

Osteoporosis is a prevalent, age-associated skeletal disorder characterized by reduced bone mass, impaired microarchitecture, and increased fragility fracture risk (1). With global population aging, osteoporosis has become a major driver of disability, mortality (2, 3), and healthcare expenditure worldwide (4, 5). Although current clinical management has substantially improved fracture prevention (6), prevailing diagnostic and therapeutic paradigms still emphasize “bone quantity” (e.g., bone mineral density) and downstream remodeling imbalance (1, 6), which can overlook upstream determinants that govern bone homeostasis and treatment responsiveness. This gap has motivated a conceptual shift from viewing bone as an isolated mineralized tissue toward recognizing it as a dynamic organ embedded within a complex, information-rich microenvironment (7, 8).

The bone microenvironment provides essential structural and biological support for normal skeletal function, and its complexity and dynamic regulation are fundamental determinants of bone health (9, 10). It comprises multiple interacting components—including bone cells, the extracellular matrix, vascular networks, and soluble mediators such as cytokines and growth factors—that together form an integrated regulatory network (11–13). Under physiological conditions, this microenvironment maintains skeletal homeostasis by tightly coordinating osteoblast-mediated bone formation and osteoclast-mediated bone resorption (14, 15). With aging, however, the microenvironment’s homeostatic capacity progressively declines, and cross-talk among bone-resident cells becomes less coordinated, creating a permissive milieu for osteoporosis development (16, 17). Importantly, deterioration of the bone microenvironment is not merely a passive consequence of aging but reflects active, multifactorial biological dysregulation (18, 19).

The pathophysiology of osteoporosis is multifaceted and influenced by numerous interacting factors (20, 21). Hormonal changes represent a central driver; in particular, the abrupt decline in estrogen after menopause accelerates bone loss and reduces bone strength (22, 23). In addition, inflammatory mediators contribute substantially to skeletal remodeling (24, 25). Chronic low-grade inflammation can activate nuclear factor-κB (NF-κB) signaling, thereby enhancing osteoclastogenesis and promoting bone resorption. Oxidative stress (26, 27) and mitochondrial dysfunction (28, 29) also play important roles, as excessive reactive oxygen species can induce osteocyte apoptosis and impair the survival and differentiation of bone-forming cells. Genetic susceptibility, nutritional status, and lifestyle factors further interact with these biological processes to shape disease onset and progression, underscoring the complexity of osteoporosis pathogenesis (30, 31).

Regulatory factors within the bone microenvironment—including cytokines, growth factors, and hormones—act as key determinants of osteoporosis by controlling bone remodeling through interrelated signaling pathways (32, 33). The receptor activator of nuclear factor-κB ligand/osteoprotegerin (RANKL/OPG) axis represents a prototypical regulatory system that governs osteoclast formation and activity and thereby maintains remodeling balance (34, 35). Bone morphogenetic proteins (BMPs) (36)matrix regulation (37), whereas pro-inflammatory cytokines such as interleukin-6 (IL-6) and tumor necrosis factor-α (TNF-α) promote osteoclast activation and bone resorption, reshaping the microenvironment toward a catabolic state (38). Maintaining the dynamic equilibrium among these mediators is essential for preserving skeletal integrity (39, 40). To provide an at-a-glance overview of major bone microenvironment regulators implicated in osteoporosis, we summarize their classification, principal sources, remodeling direction, and evidence level in **Table 1**.

**Table 1:** Bone microenvironment regulators implicated in osteoporosis: classification, sources, remodeling direction, and evidence.

Class	Regulators	Major source(s)	Direction (OP/aging)	Primary effect	Key notes/pathways	Evidence	Ref.
Osteoclastogenic axis	RANKL/OPG	Osteoblasts, stromal cells	RANKL/OPG ratio ↑	RANKL promotes osteoclast formation; OPG inhibits	Core “resorption switch”; imbalanced by inflammation	M/A/H/RCT	(34, 35, 41–46)
Pro-inflammatory cytokines	IL-6, TNF-α, IL-1	Immune cells, stromal cells	↑ (inflammaging)	↑resorption; IL-6 also ↓ osteoblast function	Drives RANKL up; forms inflammatory loop	M/H	(38, 47–53)
Wnt inhibitors	Dkk-1, SOST (sclerostin)	Osteocytes/osteoblast lineage	↓ osteoblast differentiation; ↓ bone formation	Suppress Wnt signaling; reduce formation	Upregulated in OP; suppresses Wnt/β-cat	M/A/H/RCT/B	(54, 55)
Osteogenic growth factors	BMPs, TGF-β	Bone matrix, osteoblast lineage	↓/context-dependent	↑ osteoblast differentiation/matrix synthesis	Formation-side regulators	M/A	(36, 37, 56–61)
Survival & anabolic mediators	IGF-1	Systemic + local	↓ (IGF-1 axis)	↑ osteoblast survival and differentiation	Often acts via PI3K/AKT	M/A/H	(56–62)
Hormonal factors	Estrogen (decline), PTH (context-dependent)	Endocrine system	Estrogen ↓; PTH context-dependent	Estrogen loss -> ↑ resorption; PTH context-dependent	Sets systemic “aging backdrop”	H/RCT	(20–31)
Oxidative stress mediators	ROS (oxidative stress state)	Multi-cellular	↑ (ROS)	↑ osteoblast apoptosis; ↑ osteoclast activation	Activates NF-kB/JNK; couples to inflammation	M/A/H	(23, 63–71)
Senescence program	SASP (IL-6, TNF-α etc.), PADI2 (example)	Senescent osteocytes/MSCs	↑ (senescent/SASP)	SASP -> ↑ inflammation -> ↑ resorption, ↓ formation	Aging “hub”: senescence <-> ROS <-> NF-kB	M/A/H	(72–91)
ECM/adhesion & mineralization	Collagen/proteoglycans (ECM), EphrinB2	Bone matrix, osteoblasts	ECM quality ↓; crosslinking ↑	Alters osteoblast attachment/proliferation; mineralization control	ECM remodeling changes mechanobiology	M/A	(60, 92–102)
Angiogenic niche factors	VEGF, VEGFR2 (signaling)	Endothelium/osteoblast niche	Perfusion/H-type vessels ↓; VEGF ↓	Vascular decline -> nutrient/oxygen limitation -> dysmetabolism	Links angiogenesis <-> bone turnover	M/A/H	(103–107)
“Bone-immune” communication	NF-kB (as hub)	Many cell types	↑ (NF-kB tone)	Amplifies cytokine cascade; sustains vicious cycle	Connects inflammation to Wnt/RANKL	M/A	(47–53, 85–90)
Vitamin D axis	Vitamin D activation/availability	Systemic + local	↓ (deficiency common)	Indirect: affects Ca handling; inflammation can impair activation	Bridges metabolism-inflammation	H/RCT	(108–112)

Evidence type codes — M, mechanistic (*in vitro*/*in vivo*); A, animal *in vivo*; H, human observational/clinical cohort; RCT, randomized controlled trial; B, biomarker/clinical association

Overall, the regulatory actions of bone microenvironmental factors in osteoporosis are multidimensional and operate across multiple biological levels. Beyond directly influencing osteoblast and osteoclast function (113), these mediators indirectly affect bone health by modulating intercellular communication (114, 115), inflammatory signaling (116), and oxidative stress responses (26, 117). Therapeutic strategies that precisely modulate the expression or activity of key regulators may therefore help attenuate osteoporosis progression.

In this review, we synthesize emerging evidence that osteoporosis is best understood through the lens of multi-niche microenvironment regulation and age-related microenvironment collapse. This paper summarizes the multidimensional architecture and interaction mechanisms of the cellular niche, structural/vascular niche, immune niche, and neural niche that coordinately regulate bone remodeling, and explores how aging disrupts the homeostasis of each niche through pathways such as cellular senescence, extracellular matrix and vascular degeneration and chronic immune imbalance. Meanwhile, it highlights the translational medical application prospects of targeting the bone microenvironment, including microenvironment-derived biomarkers for early diagnosis, niche-restoring therapeutic strategies, as well as novel diagnostic and treatment regimens that integrate multi-omics and artificial intelligence technologies for patient stratification and precision intervention guidance.

## 2 Multidimensional regulation of the osteogenic microenvironment

### 2.1 The cellular niche: fate determination and crosstalk

#### 2.1.1 BMSC lineage allocation and osteogenic potential

**Figure 1:**
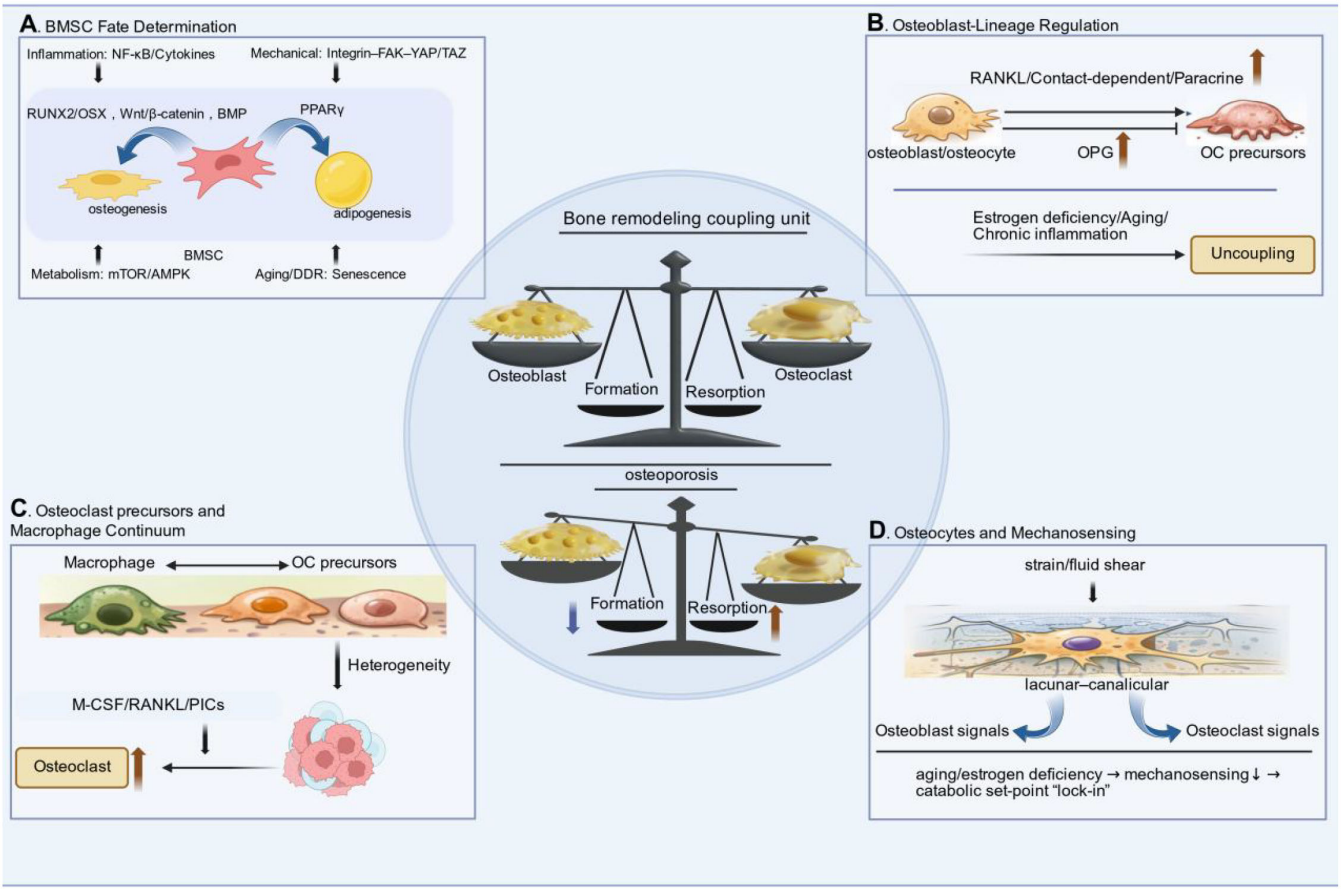
The central coupling unit depicts osteoblast (OB)–osteoclast (OC) coordination governed by the RANKL–RANK–OPG axis, which determines the remodeling set-point (formation vs resorption). **(A)** Bone marrow mesenchymal stromal/stem cells (BMSCs) exhibit a fate shift from osteogenesis toward adipogenesis under inflammatory, mechanotransductive, metabolic, and senescence-related cues, reducing osteogenic output. **(B)** Osteoblast-lineage cells (including osteoblasts/osteocytes) regulate osteoclastogenesis via RANKL/OPG and additional paracrine or contact-dependent signals; estrogen deficiency, aging, and chronic inflammation promote “uncoupling”. **(C)** Osteoclast precursors span a macrophage–osteoclast continuum with context-dependent heterogeneity, influencing precursor priming, fusion, and resorptive capacity. **(D)** Osteocytes sense mechanical strain/fluid shear through the lacunar–canalicular network and orchestrate anabolic/catabolic outputs to OBs and OCs; impaired mechanosensing in aging or estrogen deficiency locks in a catabolic set-point, accelerating net bone loss. BMSC, bone marrow mesenchymal stromal/stem cell; OB, osteoblast; OC, osteoclast; OPG, osteoprotegerin; RANKL, receptor activator of nuclear factor-κB ligand; M-CSF, macrophage colony-stimulating factor.

Bone marrow mesenchymal stromal/stem cells (BMSCs) constitute a central cellular reservoir that sustains osteoblast-lineage supply and defines the osteogenic differentiation of the microenvironment. In osteoporotic settings, BMSCs commonly display a convergent phenotype—reduced clonogenicity and osteogenic output (e.g., CFU-F frequency, ALP activity, mineralized nodule formation) accompanied by increased adipogenic commitment and marrow adipose expansion—paralleling declines in dynamic histomorphometric indices of formation (e.g., MAR/BFR) and trabecular integrity (e.g., BV/TV) *in vivo* (62, 118, 119) (**Figure 1A**). Mechanistically, lineage allocation reflects a shift in dominant transcriptional and signaling modules: attenuation of osteogenic pathways (Wnt/β-catenin, BMP–RUNX2/OSX) alongside reinforcement of adipogenic programs (PPARγ-driven networks), with additional tuning by inflammatory signaling (e.g., NF-κB-linked cytokine milieu) and mechano-metabolic inputs (integrin–FAK/YAP–TAZ, mTOR/AMPK) (56–61). Notably, whether lineage drift is primarily cell-intrinsic (senescence/DDR) or imposed by niche cues (inflammation, ECM mechanics, perfusion) likely varies by skeletal compartment and remains incompletely resolved, highlighting the need for spatially anchored, human-relevant validation.

#### 2.1.2 Osteoblast-lineage control of osteoclastogenesis

Osteoblast-lineage cells (including osteoblasts and osteocytes) act as the core coupling regulators in bone remodeling, by providing permissive regulatory signals and rate-limiting regulatory signals for osteoclast differentiation within the basic multicellular unit. The receptor activator of nuclear factor-κB ligand-receptor activator of nuclear factor-κB-osteoprotegerin (RANKL–RANK–OPG) signaling axis represents a canonical coupling node in bone remodeling. Cell-type-targeted perturbation experiments have confirmed that modulating the expression levels of RANKL/OPG in osteoblast-lineage cells is sufficient to reprogram the number and bone surface coverage of osteoclasts, thereby altering bone turnover status and trabecular architecture *in vivo* (41–43). Beyond the RANKL–OPG signaling axis, osteoblast-lineage cells can also regulate the priming of osteoclast precursors, the lifespan of mature osteoclasts, and the recruitment efficiency of osteoblasts via contact-dependent and paracrine mediators. This enables the transition from bone resorption to bone formation, ultimately maintaining bone turnover balance rather than simply suppressing the resorption process (44). In osteoporosis-relevant pathological conditions such as estrogen deficiency, aging, and chronic inflammation, this bone remodeling coupling set-point undergoes maladaptive shifts—characterized by exaggerated osteoclastogenesis concurrent with impaired osteoblast function. This effectively “uncouples” high bone resorption from compensatory bone formation, thereby accelerating net bone loss (45, 46) (**Figure 1B**). Notably, the relative regulatory contributions of coupling signals derived from osteoblasts versus osteocytes may vary across different skeletal sites and disease contexts. This highlights the necessity of conducting compartment-resolved, human-aligned validation studies on the hierarchical organization of bone coupling.

#### 2.1.3 Osteoclast precursors and the macrophage–osteoclast continuum

Osteoclasts originate from myeloid progenitors, which share overlapping developmental and functional regulatory programs with macrophages (120). Together, these two cell types form the macrophage-osteoclast continuum, through which the body’s inflammatory status and local tissue microenvironment jointly determine the ultimate level of bone resorptive function (121, 122). Osteoclast precursors are not a homogeneous cell population but exhibit detectable heterogeneity in recruitment, priming, and fusion capacity (123) (**Figure 1C**). Niche cues (e.g., the bioavailability of macrophage colony-stimulating factor/receptor activator of nuclear factor-κB ligand, pro-inflammatory cytokines, etc.) can reprogram the responsiveness of these precursors by altering their receptor expression profiles, intracellular metabolic regulatory pathways, and cell-cell fusion propensity. This, in turn, modulates the formation efficiency of multinucleated osteoclasts and the magnitude of bone resorption (e.g., the number/surface area of tartrate-resistant acid phosphatase-positive osteoclasts, resorption pit area) (123, 124). Single-cell transcriptomic and lineage-tracing studies further support that distinct precursor subsets expand or become preferentially osteoclastogenic under pathological conditions, providing a mechanistic basis for inter-individual and context-dependent variation in “high-turnover” phenotypes observed in osteoporosis and inflammaging-related bone loss (125).

#### 2.1.4 Osteocytes as orchestrators of remodeling

Osteocytes are long-lived, matrix-embedded regulatory cells that integrate mechanical and endocrine signals to coordinately regulate bone remodeling processes within the cellular niche, acting as the regulatory hub of bone tissue. Through the lacunar-canalicular network, osteocytes sense mechanical strain and fluid shear stress and convert these stimulatory signals into regulatory output signals. These signals, in turn, modulate the activity of osteoblasts and the process of osteoclastogenesis, thereby tuning bone turnover toward adaptive bone formation or resorption depending on the mechanical loading status (92). Evidence from mechanical unloading/loading model experiments and osteocyte-targeted genetic perturbation assays indicates that impairment of osteocyte mechanosensing function and its downstream signaling pathways is sufficient to attenuate the skeletal anabolic response to mechanical loading, alter dynamic bone formation indices, and affect osteoclast-related parameters, ultimately leading to the remodeling of bone microstructure (54, 126). In osteoporosis-relevant contexts such as aging and estrogen deficiency, osteocyte dysfunction—including impaired mechanosensitivity and maladaptive coupling outputs—can “lock in” a catabolic set-point where resorption is amplified while formation fails to compensate, accelerating net bone loss (127, 128) (**Figure 1D**).

### 2.2 The structural and vascular niche

#### 2.2.1 Structural–mechanical–osteocyte integration in the bone microenvironment

The extracellular matrix (ECM) in bone tissue serves as an instructive microenvironment that integrates structural organization and biochemical signal presentation functions. The composition profile and post-translational modifications of the ECM can regulate integrin binding, growth factor sequestration and release, as well as the mechanical processes of mineralization nucleation, thereby affecting the adhesion, proliferation and maturation of osteoblasts (93, 94). Matrix mechanical properties and mechanical loading further impose upstream constraints on osteogenic processes, converting stiffness and strain into transcriptional regulation via activation of mechanotransduction modules. Osteogenesis-related cells can recognize and integrate these mechanical cues through integrin–FAK/focal adhesion signaling, mechanosensitive ion channels, and mechanotranscriptional regulators (e.g., YAP/TAZ-associated programs), which ultimately converge on the expression of osteogenic genes (60, 95, 96). At the tissue scale, the lacunar-canalicular system (LCS) of osteocytes amplifies the aforementioned processes: strain-induced interstitial fluid flow generates shear stress within the canalicular network, enabling osteocytes to sense loading and transmit signals through the interconnected network, thus coordinately regulating bone remodeling (92, 97). Experimental validation of loading conditions and combined computational-imaging analyses further demonstrate that the structural characteristics of the LCS (e.g., lacunar density, canalicular connectivity) are correlated with mechanical sensitivity and its downstream remodeling outcomes (97, 98). In the context of osteoporosis and aging, microstructural degeneration and osteocyte dysfunction can impair LCS-mediated signal transmission, leading to insufficient adaptive osteogenesis and coupling imbalance (98, 99). However, it remains challenging to distinguish the effects of ECM components from covarying mechanical factors (100, 101). Therefore, an integrated research strategy combining matrix composition profiling, mechanical testing and spatial validation is required to obtain more reliable mechanistic interpretations (102).

#### 2.2.2 Angiogenesis–osteogenesis coupling and H-type vessels

Osteogenesis is tightly coupled to vascular supply: endothelial cells not only deliver nutrients and oxygen but also maintain osteoprogenitor cells and coordinate remodeling dynamics through angiocrine signaling. Studies on osteogenesis-vascular coupling have demonstrated that disruption of vascular signals can restrict osteogenic processes; conversely, enhancement of angiogenesis can boost osteoprogenitor cell activity *in vivo* and improve osteogenesis-related outcomes (103, 104). H-type vessels have attracted attention as a spatially specific vascular component. Typically composed of a population of endothelial cells highly expressing CD31 and Endomucin, these vessels are enriched in osteogenically active regions and distributed in close proximity to osteoprogenitor cell populations, thus correlating with higher levels of bone formation (103, 105). Mechanistically, endothelial cells can promote the osteogenic microenvironment by supporting the recruitment, proliferation and differentiation efficiency of progenitor cells, thereby enhancing the coupling between angiogenesis and osteogenesis (104, 106). In osteoporosis-relevant contexts, reduced perfusion and loss of H-type vessels may suppress osteogenesis and amplify the net bone loss effect induced by enhanced osteoclastogenesis (105, 107).

### 2.3 The Immune niche: osteoimmunology dynamics

#### 2.3.1 Effects of chronic inflammation on bone density

**Figure 2:**
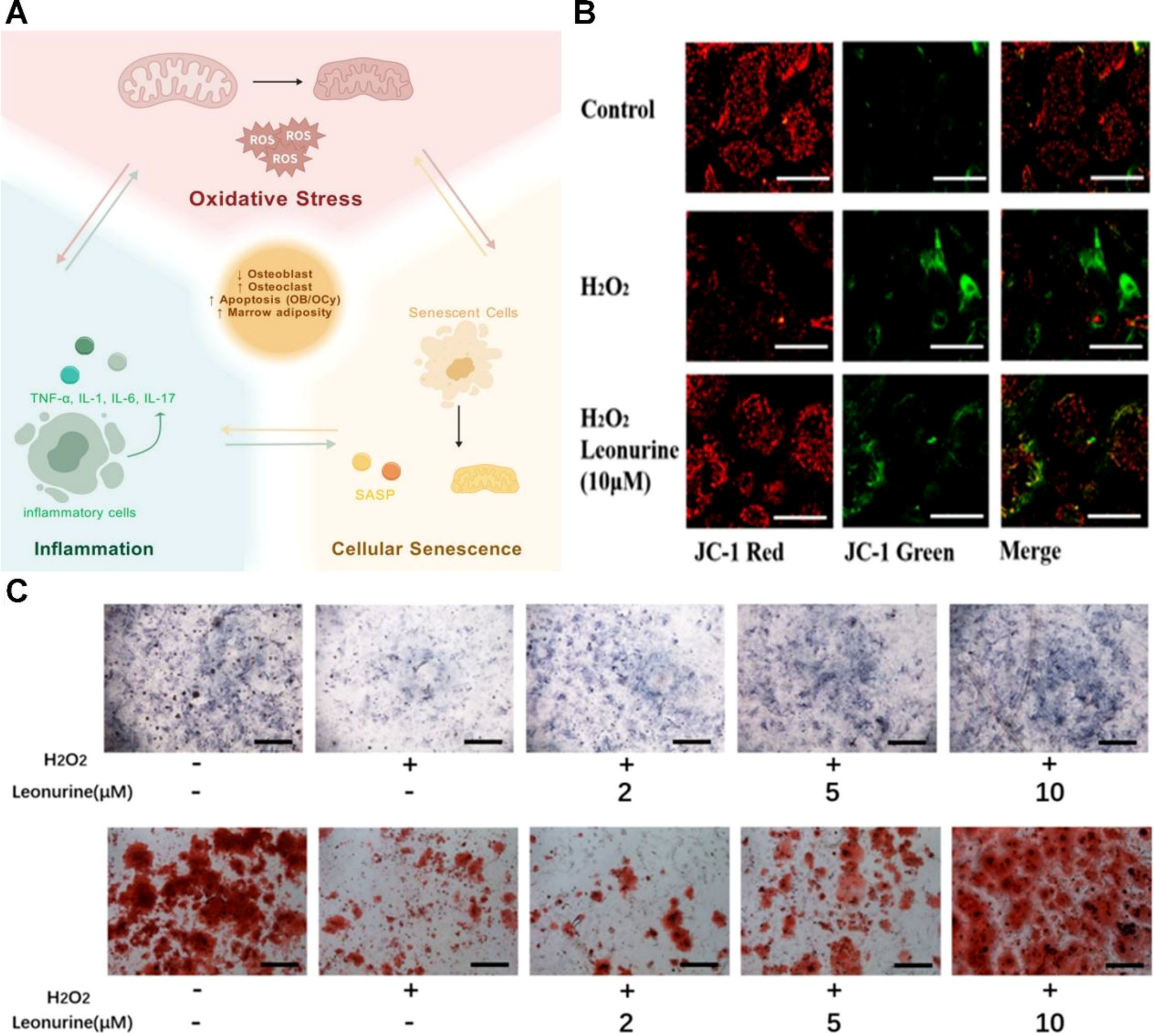
**(A)** Cellular senescence, oxidative stress, and a pro-inflammatory state can lead to increased bone resorption and decreased bone formation, disrupting bone microenvironment homeostasis and increasing the risk of osteoporosis (129). Copyright 2025, MDPI. **(B)** Reducing ROS levels through drug intervention (130). Copyright 2022, MDPI. **(C)** Protecting BMSCs from ROS damage through drug intervention, with ALP staining and Alizarin Red staining demonstrating enhanced osteogenic capacity (130). Copyright 2022, MDPI.

Chronic inflammation is widely recognized as an important contributor to osteoporosis and can markedly impair the maintenance of bone mass and bone mineral density (BMD) (108, 109). Under persistent inflammatory conditions, circulating and local levels of cytokines such as interleukin-1 (IL-1), IL-6, and tumor necrosis factor-α (TNF-α) are elevated; these mediators promote bone resorption by enhancing osteoclast differentiation and activity (**Figure 2A**). Clinical and experimental studies have reported a direct association between higher inflammatory cytokine burden and reduced BMD (110). In particular, IL-6 not only promotes osteoclastogenesis but can also suppress osteoblast function, thereby uncoupling bone formation from resorption and accelerating net bone loss. Accordingly, chronic disorders characterized by sustained inflammation—such as diabetes, rheumatoid arthritis, and chronic kidney disease—are frequently associated with lower BMD and increased fracture risk (111). Beyond direct effects on bone cells, chronic inflammation may also disrupt systemic regulators of mineral metabolism; for example, it can impair vitamin D activation (112), thereby compromising calcium absorption and utilization.

#### 2.3.2 Relationship between inflammatory cytokines and bone resorption

Inflammatory cytokines are key drivers of osteoclast activation and bone resorption in osteoporosis (47, 48). Pro-inflammatory mediators such as TNF-α and IL-1 can enhance osteoclastogenesis by upregulating RANKL expression in osteoblast-lineage and stromal cells, thereby strengthening pro-resorptive signaling (49, 50). These cytokines may also reduce osteoprotegerin (OPG) production, shifting the RANKL/OPG balance toward RANKL dominance and further amplifying bone resorption. In parallel, chronic inflammation activates nuclear factor-κB (NF-κB) signaling and promotes the sustained production of additional inflammatory mediators, creating a feed-forward cycle that reinforces osteoclast activity and progressive bone loss. IL-6 exemplifies this dual role: it contributes directly to resorption while also serving as a hallmark mediator in multiple chronic inflammatory diseases. Collectively, these mechanisms drive continuous loss of bone mass and deterioration of BMD. Therefore, therapeutic strategies that block key cytokines or modulate their downstream signaling pathways may help slow osteoporosis progression and offer clinically actionable intervention targets (51–53).

#### 2.3.3 Mechanisms of oxidative stress

Oxidative stress arises from an imbalance between reactive oxygen species (ROS) generation and antioxidant defense systems and plays a substantial role in the pathogenesis of osteoporosis (23, 63). Excess ROS can induce macromolecular damage, trigger apoptosis, and amplify inflammatory signaling. Accumulating evidence indicates that oxidative stress directly promotes osteoblast apoptosis while enhancing osteoclast differentiation and activity, thereby accelerating bone loss (64–66). Consistent with this, patients with osteoporosis often exhibit elevated circulating or tissue markers of oxidative stress, supporting a close link between redox imbalance and dysregulated bone metabolism.

Mechanistically, oxidative stress influences bone remodeling through multiple pathways. ROS can activate stress- and inflammation-related signaling cascades, including NF-κB and c-Jun N-terminal kinase (JNK), which promote the production of pro-inflammatory mediators and further stimulate osteoclastogenesis (67–69). Oxidative stress also impairs osteogenic potential at the progenitor level: beyond inducing apoptosis in osteoblasts, it can compromise bone marrow mesenchymal stem/stromal cell (BMSC) function and shift lineage commitment toward adipogenesis at the expense of osteogenesis, reducing overall bone-forming capacity (70, 71) (**Figures 2A, B**). Emerging evidence suggests that alleviating oxidative stress may serve as an adjunctive strategy for the prevention and management of osteoporosis. Interventional approaches that reduce reactive oxygen species (ROS) burden—such as antioxidant supplementation or strategies to enhance the endogenous antioxidant system—may help restore redox homeostasis and preserve skeletal health (**Figure 2C**).

## 3 Age-related deterioration: the microenvironment under siege

### 3.1 Cellular senescence and stem cell exhaustion

#### 3.1.1 SASP as a central driver of inflammaging and uncoupled bone remodeling

Cellular senescence is increasingly recognized as a core hallmark of organismal aging and a key biological process contributing to age-associated tissue dysfunction, including musculoskeletal decline (131, 132). In the bone microenvironment, the accumulation of senescent cells—particularly within bone marrow stromal cells (BMSCs), osteoblast-lineage cells, and osteocytes—has been implicated in osteoporosis pathogenesis and age-related bone loss (133–135). With advancing age, persistent stressors such as oxidative stress and mitochondrial dysfunction activate DNA damage responses (DDR) and enforce stable cell-cycle arrest, thereby reducing the proliferative capacity and osteogenic potential of BMSCs and osteoblasts (136–138).Recent high-resolution single-cell atlases have begun to resolve aging-associated niche remodeling at cellular and intercellular-communication levels, identifying senescent-like mesenchymal subclusters alongside age-biased immune populations and altered cell–cell signaling within cranial skeletal stem cell niches, supporting a direct link between senescence programs and niche dysfunction during skeletal aging (139). A defining feature of senescent cells is the acquisition of the senescence-associated secretory phenotype (SASP), a pro-inflammatory and matrix-modulating secretome that includes cytokines such as interleukin-6 (IL-6), tumor necrosis factor-α (TNF-α), and interleukin-1β (IL-1β) (133, 140). Mechanistically, SASP output is maintained by inflammatory transcriptional programs, with nuclear factor-κB (NF-κB) serving as a central hub that amplifies cytokine production and sustains a chronic inflammatory milieu (55, 141). Integrative analyses combining scRNA-seq with bulk transcriptomics and cell–cell communication inference further suggest that aging bone marrow is characterized by coordinated shifts in stromal and myeloid compartments (e.g., expansion of BMSCs and macrophages), enrichment of fibrotic and immune-inflammatory programs, and directional paracrine signaling from aged BMSCs that can promote myeloid aging-like phenotypes, collectively reinforcing inflammaging and impaired osteogenesis (142). Consistent with this concept, mechanistic work has uncovered a mechanoinflammatory autocrine loop in BMMSCs whereby loss of Piezo1 signaling enhances Ccl2/CCR2 activation, triggers NF-κB–dependent Lcn2 production, and biases BMSCs toward adipogenesis at the expense of osteogenesis, providing a concrete pathway linking inflammation, cell-fate drift, and osteoporotic bone loss (143).

**Figure 3:**
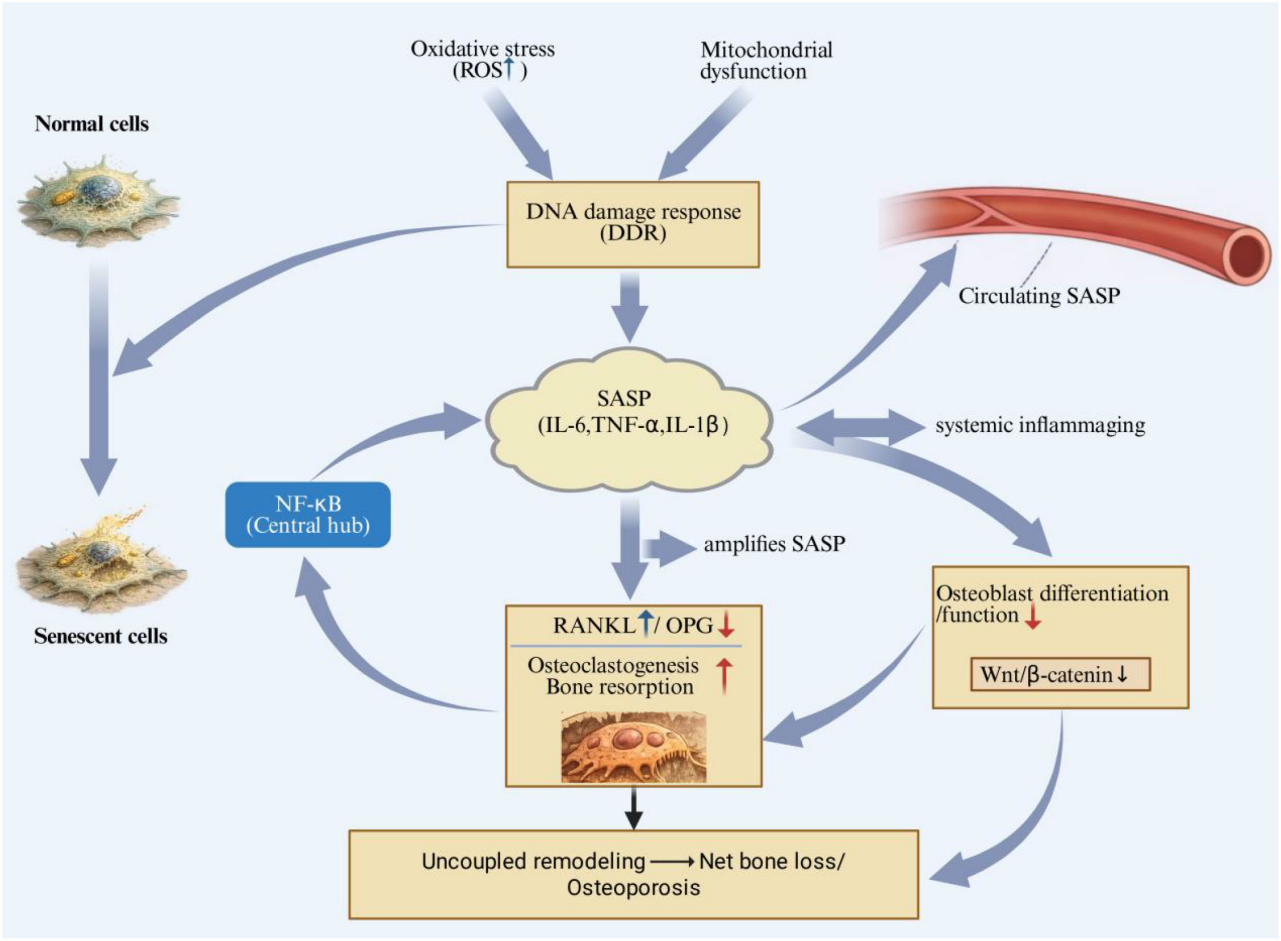
Aging-associated stressors, including oxidative stress and mitochondrial dysfunction, induce DNA damage responses (DDR) and stable cell-cycle arrest in BMSCs, osteoblast-lineage cells, and osteocytes, leading to cellular senescence. Senescent cells acquire a SASP characterized by pro-inflammatory cytokines (IL-6, TNF-α, IL-1β), which is amplified by NF-κB signaling. SASP promotes osteoclastogenesis by enhancing RANKL-dependent signaling and disturbing the OPG/RANKL balance, while impairing osteoblast function and suppressing osteoanabolic pathways, resulting in uncoupled remodeling and net bone loss. Circulating SASP factors contribute to systemic inflammaging.

In bone, a SASP-enriched environment promotes osteoclastogenesis by enhancing RANKL-dependent signaling and disrupting the OPG/RANKL balance, thereby increasing bone resorption (135, 144). Concurrently, SASP-associated inflammation impairs osteoblast differentiation and function and can suppress osteoanabolic pathways (e.g., via inhibition of Wnt signaling), culminating in uncoupled remodeling characterized by increased resorption and decreased formation (145) (**Figure 3**). Beyond local effects, circulating SASP factors contribute to systemic “inflammaging,” potentially exacerbating osteoporosis progression and increasing susceptibility to age-related comorbidities (132, 146). Importantly, targeting senescent cells has shown therapeutic promise in skeletal aging; for example, clearance of senescent cells prevented age-related bone loss *in vivo*, supporting senescence as an actionable driver rather than merely a correlate of aging (135). Recent syntheses further consolidate these mechanistic links and summarize emerging senotherapeutic strategies across skeletal pathophysiology (147). Together, senescent cells and their SASP represent key mechanistic mediators of osteoporotic bone loss and compelling targets for future interventions.

#### 3.1.2 Lineage drift: osteogenic-to-adipogenic reprogramming in the aged marrow

A prominent hallmark of skeletal aging is the lineage shift of bone marrow mesenchymal stromal/stem cells (BMSCs): the osteogenic differentiation capacity declines, accompanied by enhanced adipogenic differentiation and expansion of bone marrow adipose tissue (148, 149). At the transcriptional regulatory level, osteogenic modules such as RUNX2/OSX, which are driven by the Wnt/β-catenin and BMP pathways, become less amenable to activation. In contrast, the adipogenic regulatory network centered on PPARγ and C/EBP family members predominates, thereby lowering the threshold for adipogenic differentiation and raising the barrier to osteogenic initiation (61, 150). This cellular reprogramming is not entirely governed by cell-intrinsic factors but is subject to persistent regulation by the aging microenvironment. Inflammatory signals enriched in the senescence-associated secretory phenotype (SASP) and the NF-κB-dependent cytokine milieu can suppress pro-osteogenic pathways and enhance the sensitivity of precursor cells to adipogenic cues. Concurrently, mechano-metabolic inputs—including reduced mechanical loading, altered ECM mechanical properties, and changes in the activity of pathways such as AMPK/mTOR and YAP/TAZ—further skew the differentiation fate of BMSCs from osteogenesis toward adipogenesis (57, 60, 151). Mounting evidence also indicates that BMSC populations themselves exhibit inherent heterogeneity. Aging can amplify the lineage shift by altering the proportion and plasticity of distinct precursor subpopulations (e.g., expansion of certain subpopulations, pre-activation of adipogenic programs, or loss of osteogenic potential). This provides a mechanistic explanation for the interindividual variability in bone marrow fat accumulation and osteogenic decline observed in aging populations (152, 153). In terms of functional outcomes, the osteogenic-to-adipogenic drift limits the supply of osteoblast-lineage cells and favors a “resorption-over-formation” remodeling pattern. This accelerates net bone loss and impairs the integrity of bone microstructure (148, 149).

### 3.2 ECM stiffening and vascular rarefaction

**Figure 4:**
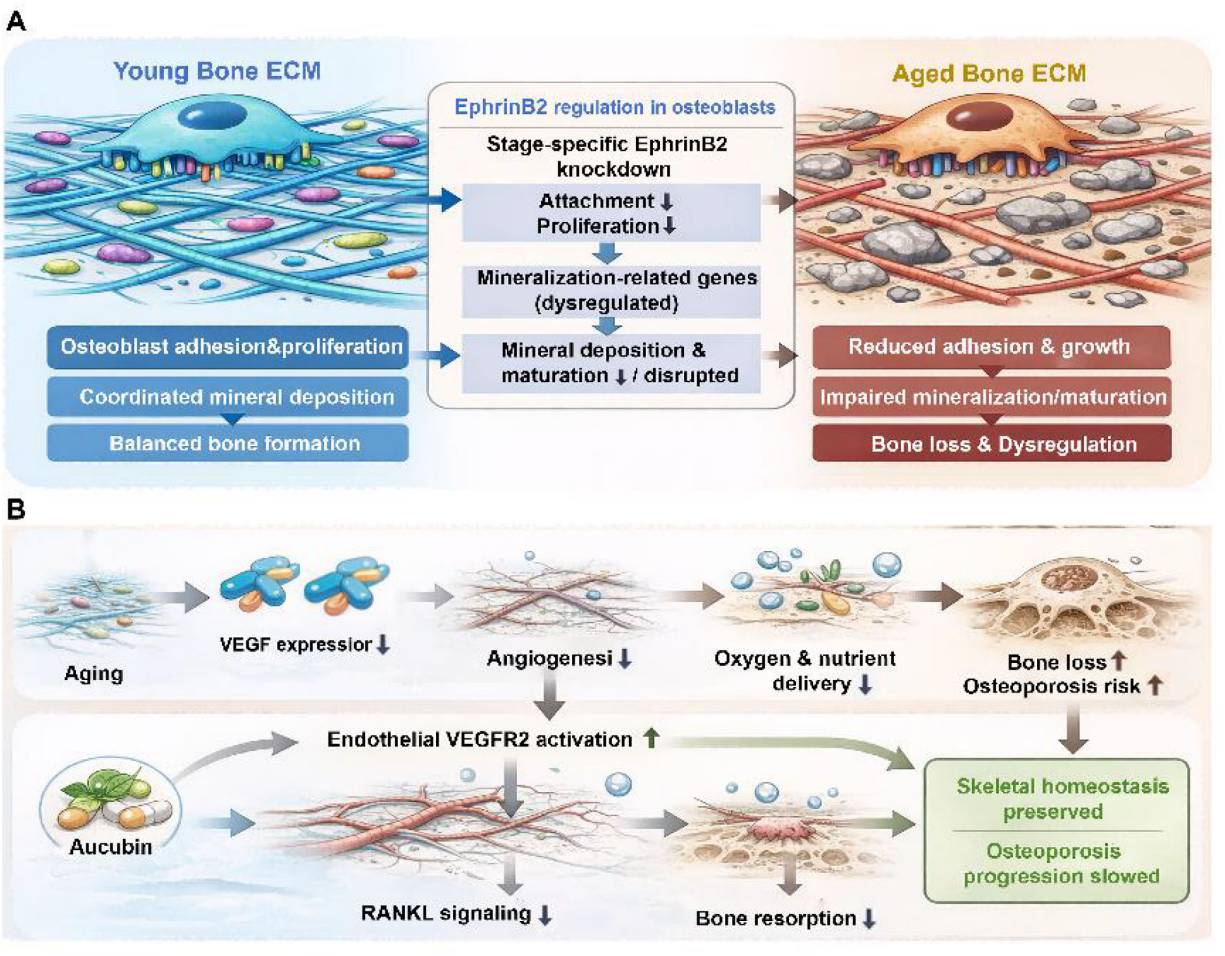
**(A)** Aging-associated extracellular matrix (ECM) remodeling impairs osteoblast adhesion and disrupts mineralization maturation via EphrinB2-dependent regulation. **(B)** Age-related decline in angiogenic capacity synergistically induces vascular insufficiency and bone loss, whereas aucubin exerts dual protective effects by enhancing VEGFR2 expression to promote angiogenesis and inhibiting RANKL expression to reduce bone resorption.

Aging is also accompanied by remodeling of the extracellular matrix (ECM), which is critical for maintaining bone microenvironment function (154, 155). With advancing age, the composition and organization of collagen and proteoglycans change, altering the matrix’s mechanical properties and bioactivity. Such ECM remodeling can impair osteoblast adhesion, proliferation, and function, and may weaken osteoblast control over mineralization (e.g., by disrupting matrix cues that coordinate deposition and maturation of mineral). Blank and colleagues investigated osteoblast regulation using stage-specific EphrinB2 knockdown, demonstrating that EphrinB2 is required for osteoblast attachment and proliferation and dynamically regulates mineralization. These findings suggest that EphrinB2 not only influences osteoblast differentiation and function but also contributes to mineralization maturation by modulating the expression of mineralization-related genes (156) (**Figure 4A**).

In addition to matrix alterations, aging is associated with diminished angiogenic capacity and reduced vascular supply within bone (157, 158). Expression of angiogenesis-related factors, including vascular endothelial growth factor (VEGF), declines with age, which can limit oxygen and nutrient delivery and further exacerbate metabolic dysregulation in skeletal tissue. Impaired vascularization reduces nutrient availability within the bone microenvironment and thereby facilitates osteoporosis development (159, 160) (**Figure 4B**). He and colleagues highlighted a dual role for aucubin in this context, showing that it promotes VEGFR2-mediated angiogenesis—improving microenvironmental nutrient support—while simultaneously attenuating RANKL-induced bone resorption, collectively slowing osteoporosis progression. This work underscores the tight coupling between angiogenesis and bone metabolism and highlights effective vascularization as an important determinant of skeletal homeostasis and protection against bone loss (161).

In summary, aging exerts multifactorial effects on bone homeostasis through coordinated changes in gene expression and epigenetic regulation, cellular senescence, ECM remodeling, and declines in angiogenic capacity. Defining how these processes interact will be essential for developing preventive and therapeutic strategies for age-related osteoporosis and for improving skeletal health in older adults (162–164).

### 3.3 “Inflammaging”: the chronic immune shift

.The bidirectional interplay between cellular senescence and inflammation is increasingly recognized as a driver of skeletal aging and osteoporosis, rather than a secondary epiphenomenon (165, 166). Senescent bone-resident cells can sustain a chronic, low-grade inflammatory state through the senescence-associated secretory phenotype (SASP), which is enriched in pro-inflammatory cytokines (e.g., IL-6, TNF-α, and IL-1 family members) and matrix-modulating factors (140, 165). Mechanistically, persistent DNA-damage signaling can maintain inflammatory cytokine secretion in senescent cells, providing a molecular basis for long-lasting SASP output (136). Among SASP regulatory nodes, NF-κB has been identified as a central transcriptional hub that amplifies and stabilizes inflammatory secretome programs in senescence (55). In the skeletal context, NF-κB signaling is also a key determinant of remodeling balance: it is required for osteoclastogenesis and inflammatory bone resorption while it can impair osteoblast differentiation and osteoanabolic signaling (including crosstalk with Wnt/β-catenin pathways) (72, 73).

**Figure 5:**
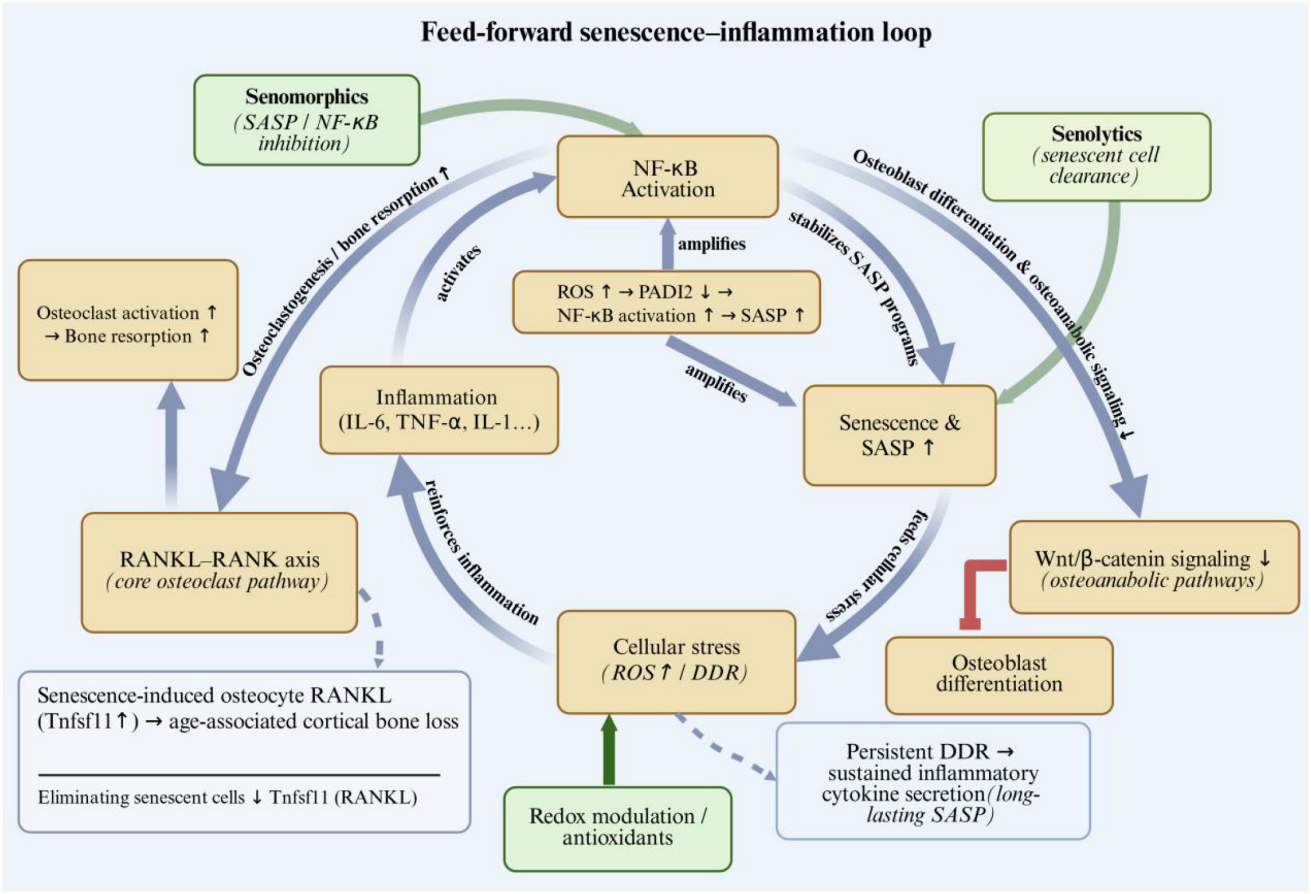
Senescence and inflammation form a feed-forward loop in aging bone, in which persistent DDR sustains inflammatory cytokine secretion, and NF-κB integrates inflammatory signals to promote osteoclast-mediated resorption and suppress osteoblast differentiation/osteoanabolic signaling. Senescence-induced osteocyte RANKL (Tnfsf11) contributes to age-related cortical bone loss, and eliminating senescent cells reduces RANKL expression in cortical bone. Oxidative stress further amplifies SASP production via a ROS–PADI2–NF-κB axis. Therapeutic interruption of this network (senolytics, senomorphics targeting SASP/NF-κB, and redox-modulating approaches) may help rebalance bone remodeling.

This establishes a feed-forward loop in the aging bone microenvironment: inflammation and cellular stress promote senescence, which in turn increases SASP burden and further reinforces inflammatory signaling (55, 140, 166) (**Figure 5**). At the remodeling level, pro-inflammatory cues converge on the RANKL–RANK axis, a core pathway controlling osteoclast differentiation and activation (74, 120). Importantly, age-associated cortical bone loss has been mechanistically linked to increased osteocyte-derived RANKL that is induced by senescence; notably, eliminating senescent cells reduced Tnfsf11 (RANKL) expression in cortical bone, providing direct evidence that senescence can be upstream of a RANKL-driven pro-resorptive shift (135, 144). Oxidative stress can further potentiate this circuitry; for example, ROS-driven osteoblast senescence has been mechanistically connected to enhanced SASP production via NF-κB activation through a ROS–PADI2–NF-κB axis (138). Collectively, these findings support therapeutic strategies that interrupt the senescence–inflammation network (e.g., senolytics, senomorphics targeting SASP/NF-κB, and redox-modulating approaches) to rebalance remodeling and mitigate osteoporosis progression (135, 166).

### 3.4 Gene expression regulation and epigenetic modifications

**Figure 6:**
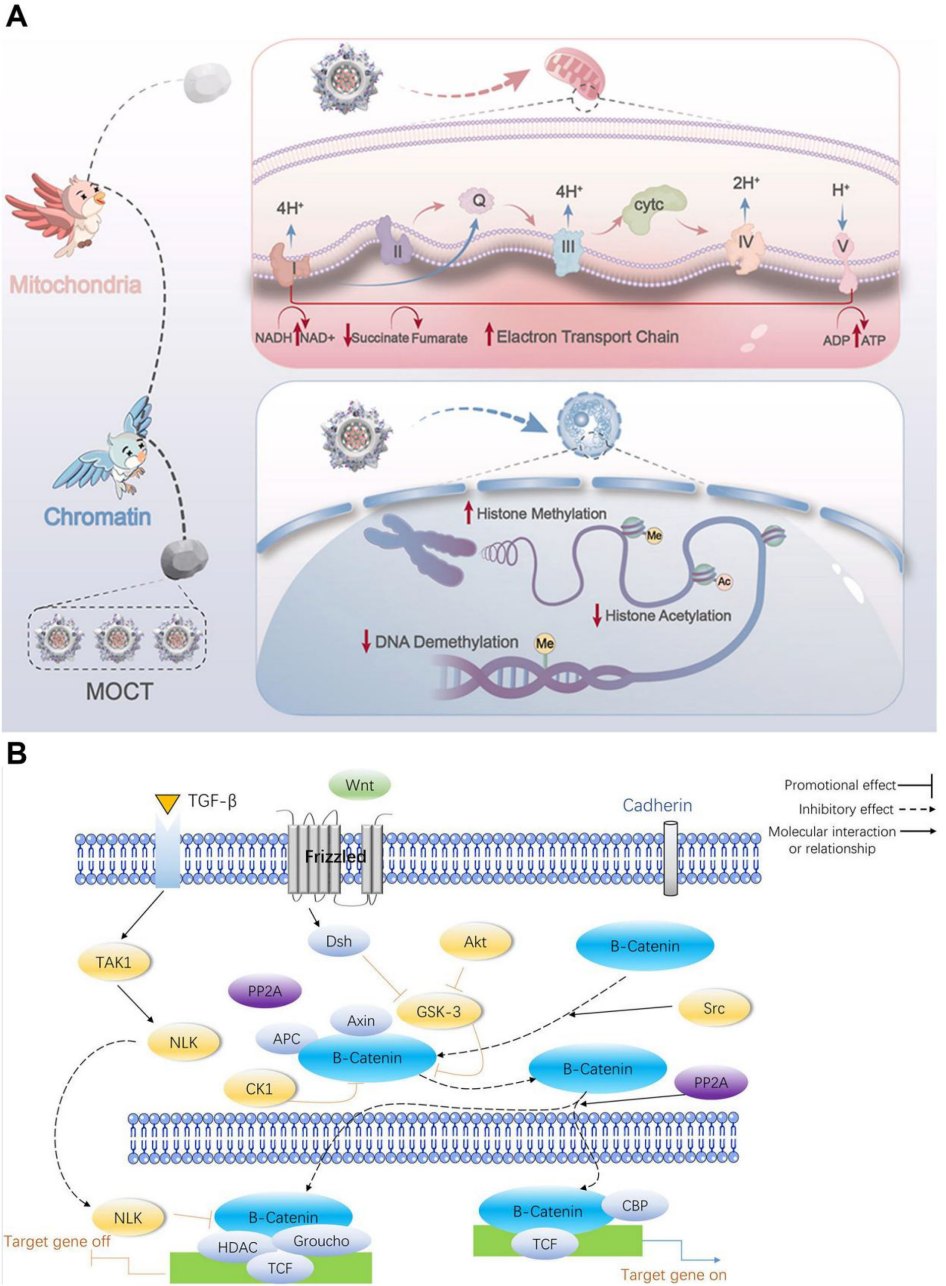
**(A)** Activating the PI3K/Akt/mTOR pathway promotes autophagy, thereby protecting bone marrow mesenchymal stem cells from oxidative stress injury (84). Copyright 2026, ELSEVIER SCI LTD. **(B)** A relatively small proportion of osteocytes become senescent under stress stimuli. These cells likely induce an inflammatory microenvironment in the bone via SASP secretion, disrupting bone formation and enhancing osteoclast function (85).

Aging is accompanied by profound shifts in gene expression, and the abundance of many bone microenvironment regulators in skeletal tissues changes in an age-dependent manner (75, 76). For example, age-related osteoblast dysfunction is often associated with decreased expression of osteogenic growth factors, including bone morphogenetic proteins (BMPs) and transforming growth factor-β (TGF-β). Such changes can directly impair osteoblast proliferation and differentiation and may also destabilize the bone microenvironment by perturbing feedback networks that normally maintain remodeling homeostasis (77) (**Figures 6A, B**).

Mechanistic studies further underscore how altered signaling and chromatin regulation reshape the aged bone niche. Li et al. reported that modulation of PDGF-AA downregulated PDGFRα, thereby relieving repression of the BMP–Smad1/5/8 pathway and enhancing mesenchymal stem cell (MSC) osteogenic differentiation and migration; this was accompanied by reduced TGF-β signaling, collectively influencing bone microenvironment homeostasis (78). Similarly, Sinha and colleagues showed that Pbrm1 promotes PBAF-dependent chromatin remodeling to sustain BMP and TGF-β expression, thereby preserving osteogenic capacity and microenvironmental stability. In contrast, Pbrm1 loss reduced these key signals and impaired bone regeneration and repair (79).

In parallel, aging-associated epigenetic remodeling—including DNA methylation and histone modifications—modulates the transcriptional programs that govern the production and activity of bone microenvironment regulators (80, 81). Ullah et al. demonstrated that knockout of α-1,3-galactosyltransferase was associated with altered DNA methylation patterns, reduced MSC proliferation and differentiation, accelerated cellular senescence, and disruption of bone microenvironment homeostasis (82). Moreover, epigenetic regulation of cell-cycle–related genes appears to contribute to age-associated declines in osteoblast proliferative capacity and increased senescence (83). For example, Varela and colleagues reported that age-dependent epigenetic regulation of ZNF687—linked to miR-142a-3p and DNA methylation—downregulated key cell-cycle genes, thereby impairing osteoblast proliferation and accelerating cellular senescence and deterioration of bone metabolic function (81).To integrate the convergent mechanisms discussed above, **Table 2** maps major signaling axes in osteoporosis to aging-related triggers, remodeling outcomes, and clinically actionable target nodes.

**Table 2:** Key signaling axes in osteoporosis: triggers, remodeling outcomes, and targetable nodes.

Axis/Table 1 class	Aging triggers	Main outcome	Key nodes	Targetability	Interventions & readouts	Ref.
Wnt/beta-catenin suppression (Class: Wnt inhibitors)	Aging suppression; inflammation (NF-kB)	↓ osteoblast differentiation -> ↓ formation	β-catenin; Frizzled; Dkk-1; SOST	High	Interventions: Anti-sclerostin; Dkk-1 blockade (experimental); Wnt restoration strategies; Readouts: BMD; osteogenic markers; Dkk-1/SOST levels	(54–61)
RANKL-RANK-OPG imbalance (Class: Osteoclastogenic axis)	IL-6/TNF-α up -> RANKL↑; OPG↓	↑ osteoclast formation/activation -> ↑ resorption	RANKL, RANK, OPG	High	Interventions: Anti-RANKL; OPG-mimetic antagonism; downstream osteoclast blockade; Readouts: CTX/TRAP5b; RANKL/OPG ratio	(34, 35, 41–46)
PI3K/AKT-mTOR/autophagy (Class: Survival & anabolic mediators)	Reduced pathway activity in OP; growth factors	↓ osteoblast survival/proliferation	PI3K, AKT; IGF-1 (upstream)	Medium	Interventions: IGF-1/PI3K support; metabolic tuning (AMPK/mTOR); autophagy modulation; Readouts: Osteoblast survival; mineralization markers	(56–62)
NF-kB inflammatory hub (Class: Pro-inflammatory cytokines)	Chronic low-grade inflammation	↑ osteoclastogenesis; vicious cycle	NF-kB; IL-6; TNF-α	Medium	Interventions: Anti-cytokine approaches; NF-kB pathway dampening (preclinical); combination with anabolic support; Readouts: CRP/cytokines; osteoclast markers	(47–53, 85–90)
Oxidative stress->NF-kB/JNK (Class: Oxidative stress mediators)	ROS accumulation, mitochondrial dysfunction	↑ osteoblast apoptosis; ↑ osteoclast activity	ROS; NF-kB; JNK	Medium	Interventions: Antioxidant/Nrf2 activation; mitochondrial protection; anti-inflammatory synergy; Readouts: Oxidative stress markers; BMD	(23, 63–71)
Senescence/SASP (Class: Senescence program)	Aging; ROS-driven senescence	SASP->inflammation amplification	SASP; IL-6; TNF-α	Emerging	Interventions: Senolytics; senomorphics/SASP suppression; JAK/NF-kB dampening (context-dependent); Readouts: Senescence markers; cytokine panels	(72–91)
ECM remodeling & stiffness shift (Class: ECM/adhesion & mineralization)	Collagen/proteoglycan changes with age	Impaired adhesion, proliferation, mineralization	ECM components; EphrinB2	Medium-Low	Interventions: Matrix-quality targeting (crosslinking/ECM turnover); mechanical loading; TGF-β tuning (context-dependent); Readouts: Histomorphometry; matrix markers	(60, 92–102)
Angiogenesis decline & perfusion loss (Class: Angiogenic niche factors)	VEGF down; vascular niche impairment	Nutrient/oxygen deficit -> dysmetabolism	VEGF; VEGFR2	Medium	Interventions: Pro-angiogenic strategies; exercise/loading; endothelial-osteogenic coupling support; Readouts: Vessel density; VEGF levels	(103–107)
Inflammation-Wnt crosstalk (Class: Cytokines + Wnt inhibitors)	Cytokines suppress Wnt	“Formation brake” strengthened	NF-kB <-> Wnt	Medium (combo)	Interventions: Dual modulation: cytokine control + Wnt restoration; Readouts: Combined cytokine + Wnt inhibitor profiling	(47–61)
Inflammation-RANKL crosstalk (Class: Cytokines + RANKL axis)	TNF-α/IL-1 upregulate RANKL	“Resorption accelerator”	TNF-α/IL-1 -> RANKL	High	Interventions: Anti-cytokine + anti-RANKL; restore OPG/RANKL balance; Readouts: RANKL, CTX	(34, 35, 38, 41–53)

Targetability is an editorial synthesis (High/Medium/Low/Emerging) based on clinical tractability and safety constraints; readouts are representative examples.

## 4 Interventions and treatment strategies for osteoporosis

Treatment paradigms for osteoporosis are shifting from single-agent therapy toward comprehensive, personalized, multi-target approaches (86, 87). Rapid advances in genomics and proteomics have expanded the scope of precision medicine, enabling treatment plans to be tailored according to a patient’s genetic background, skeletal metabolic profile, and inflammatory status. In parallel, cell-based strategies are gaining momentum, with mesenchymal stem/stromal cell therapies and exosome-based interventions emerging as major areas of investigation. Rather than targeting a single molecule, these approaches aim to restore bone homeostasis through broader regulation of the bone microenvironment (88, 89).

For example, Wang et al. analyzed differentially expressed long non-coding RNAs (lncRNAs) in bone marrow MSC–derived exosomes from postmenopausal osteoporosis patients and constructed lncRNA–miRNA–mRNA interaction networks to delineate pathways relevant to bone metabolism and cellular function. Their findings underscore the value of multi-omics analyses for mechanistic discovery and inform the development of individualized therapeutic strategies (90). Moreover, gene-editing technologies such as CRISPR–Cas9 offer opportunities for targeted modulation of genes critical to bone metabolism and may ultimately enable disease-modifying interventions (91). In this context, Guo and colleagues applied dynamic network biomarker analysis to identify CDKN1A as a key factor during the initiation phase of mineralization and suggested that CRISPR–Cas9 could provide a tool to modulate pivotal genes in bone metabolism, supporting new avenues for precision therapeutics in osteoporosis (167).

Looking ahead, combining pharmacologic and non-pharmacologic interventions is likely to become a defining feature of osteoporosis management. Beyond established antiresorptives such as bisphosphonates, newer biologics—including monoclonal antibodies—have shown substantial potential to suppress bone resorption and reduce fracture risk (168–170). At the same time, personalized nutritional strategies, structured exercise and rehabilitation programs, and lifestyle modifications remain essential components of comprehensive care. The integration of artificial intelligence (AI) and large-scale clinical data may further improve early risk stratification and individualized intervention. For instance, recent work combining multiple machine-learning models (including feature selection and ensemble methods) with explainable AI approaches (e.g., SHAP and LIME) reported an osteoporosis risk-prediction system with 89% accuracy, enabling identification of high-risk individuals and supporting clinical decision-making (171). Nanotechnology-based platforms for targeted drug delivery and bone repair likewise offer promising translational directions (172, 173). Mora-Raimundo and colleagues, for example, used mesoporous silica nanoparticles to co-deliver small interfering RNA (siRNA) and osteostatin, improving bone microarchitecture and supporting tissue restoration in experimental osteoporosis models (174). Overall, future strategies will increasingly emphasize multidisciplinary collaboration, integrating advances from molecular biology, clinical medicine, nutrition, rehabilitation, and data science to achieve more precise and effective osteoporosis prevention and treatment (175, 176).To facilitate translation from mechanism to practice, **Table 3** summarizes microenvironment-targeted intervention strategies for osteoporosis, organized by evidence ladder and translational readiness.

**Table 3:** Microenvironment-targeted interventions for osteoporosis: evidence ladder and translational readiness.

Strategy	Examples	Stage	Microenvironment target	Strengths	Bottlenecks	Ref.
Classical anti-resorptives	Bisphosphonates	Approved (clinical)	Osteoclast activity Best-fit: High-turnover OP; fracture prevention	Robust efficacy, standard-of-care	Long-term safety, adherence	(2, 6, 79)
Biologics (mAbs)	Monoclonal antibodies (e.g., anti-RANKL; anti-sclerostin)	Approved (clinical)	RANKL/OPG; Wnt inhibitor axis Best-fit: Very high fracture risk; high turnover or low formation	High potency; mechanism-specific; rapid fracture-risk reduction	Cost; injection logistics; patient selection; agent-specific safety signals	(34, 35, 54, 85–87)
Cell therapy	Mesenchymal stem cell (MSC) therapy	Early clinical/preclinical	Whole microenvironment regulation Best-fit: Refractory/low-turnover niche impairment (experimental)	Multi-target, regenerative rationale	Consistency, safety, scalability	(118, 143, 144)
Exosome-based	MSC-derived exosomes; exosomal lncRNA networks	Preclinical/early	Paracrine regulation Best-fit: Regenerative niche tuning; adjunct (preclinical)	Cell-free, potentially safer	Standardization, dosing, biodistribution	(19, 80, 81)
Gene editing	CRISPR-Cas9 targeting key genes	Preclinical	Fundamental pathway control Best-fit: Monogenic/rare OP; target validation (preclinical)	Potentially disease-modifying	Delivery, off-target, ethics/regulation	[]
Network biomarker-guided targeting	Dynamic network biomarker	Preclinical (concept)	Early-phase trigger nodes Best-fit: Heterogeneous OP; early-phase trigger detection	Precision strategy	Validation + clinical translation pathway	(84)
AI risk prediction & decision support	ML + SHAP/LIME	Translational (implementation)	Early screening & intervention timing Best-fit: Population screening; individualized decisions	Scalable, preventive focus	Data bias, external validation, regulation	(88)
Nanotechnology drug delivery	Mesoporous silica nanoparticles (MSNs) delivering siRNA/osteostatin; targeted delivery	Preclinical -> translational	Targeted microenvironment delivery Best-fit: Local delivery when systemic limits	Improves local efficacy, reduces systemic toxicity	Safety, manufacturing, biodistribution	(78, 89–91)
Lifestyle & rehab	Exercise rehab, lifestyle modification	Clinical (practice)	Systemic inflammatory/metabolic milieu Best-fit: Adjunct across stages; overall health	Low cost, broad benefit	Compliance, personalization	(6, 54, 126, 127, 152)
Precision medicine/multi-omics	Genomics/proteomics-informed stratification	Emerging	Patient stratification Best-fit: Endotype-driven stratification; responder selection	Better responder identification	Cost, workflow integration	(82, 83, 125)

## 5 Conclusion and discussion

Although substantial progress has been made in elucidating how bone microenvironmental factors contribute to osteoporosis pathogenesis, important limitations remain. First, osteoporosis is a multifactorial disorder shaped by complex interactions among genetic susceptibility, environmental exposures, nutritional status, and lifestyle factors. Second, while emerging technologies—including gene editing and stem/stromal cell–based therapies—offer promising therapeutic avenues, their translation requires rigorous evaluation of long-term efficacy, safety, and feasibility. Future research should therefore strengthen the integration of basic and clinical studies to accelerate mechanistic discovery while ensuring patient safety.

Looking ahead, increasingly precise strategies for osteoporosis prevention and treatment are likely to emerge from ongoing technological advances. Precision medicine may enable treatment plans tailored to an individual’s genetic background, lifestyle, and nutritional profile. High-resolution approaches such as single-cell sequencing and proteomics can facilitate the identification of patient-specific biomarkers, supporting earlier risk stratification, screening, and targeted intervention. In parallel, the integration of artificial intelligence (AI) may improve analytical efficiency and enable the discovery of therapeutic targets from large-scale datasets, thereby refining prevention and treatment algorithms. In addition, nanotechnology and smart biomaterials provide opportunities for more efficient drug delivery and bone repair. Collectively, these innovations may shift clinical paradigms from predominantly symptomatic management toward earlier, preventive, and individualized care.

Bone microenvironmental factors are central to the initiation and progression of osteoporosis. As a dynamic tissue, bone undergoes continuous remodeling that depends on coordinated interactions among osteoblasts, osteoclasts, and other niche components. Accordingly, research should move beyond isolated single-factor models and instead interrogate multilevel, multifactorial networks that govern remodeling under diverse physiological and pathological conditions. Such integrative investigation may uncover previously unrecognized therapeutic targets and improve our ability to modulate remodeling balance. Moreover, because bone microenvironment health is closely linked to systemic physiology, insights into microenvironmental dysregulation may also inform studies of other bone-related disorders.

In summary, osteoporosis is a complex, multifactorial disease driven in part by dysregulation of bone microenvironmental factors. A comprehensive understanding of these regulators and their interactions can reveal actionable intervention targets and guide future research priorities. Coupled with emerging technologies, bone microenvironment–focused research is expected to advance early screening and personalized management of osteoporosis. Continued progress will require multidisciplinary, systems-oriented approaches that bridge basic mechanisms with clinical translation, ultimately improving skeletal health and quality of life.
